# A generative AI multi-agent framework with integrated XAI governance for cancer diagnostics: from multi-omics interpretation to lifestyle risk stratification

**DOI:** 10.3389/fsysb.2026.1892418

**Published:** 2026-08-03

**Authors:** Chamseddine Barki, Mariem Chouchen, Afef Sediri, Halil İbrahim Ceylan, Hanene Boussi Rahmouni, Raul Ioan Muntean, Hesham R. El-Seedi, Nicola Luigi Bragazzi, Ismail Dergaa

**Affiliations:** 1 Research Laboratory of Biophysics and Medical Technologies, The Higher Institute of Medical Technologies of Tunis, University of Tunis El Manar, Tunis, Tunisia; 2 Department of Physical Education and Sports Teaching, Faculty of Sports Sciences, Atatürk University, Erzurum, Türkiye; 3 The Computer Science Research Centre, University of the West of England, Bristol, United Kingdom; 4 Department of Physical Education and Sport, Faculty of Law and Social Sciences, University “1 Decembrie 1918” of Alba Iulia, Alba Iulia, Romania; 5 Department of Chemistry, Faculty of Science, Islamic University of Madinah, Madinah, Saudi Arabia; 6 Department of Clinical Pharmacy, Saarland University, Saarbrücken, Germany; 7 High Institute of Sport and Physical Education of Ksar Said, University of Manouba, Manouba, Tunisia; 8 High Institute of Sport and Physical Education of El Kef, University of Jendouba, Jendouba, Tunisia

**Keywords:** agentic AI, cancer diagnostics, cancer risk stratification, explainable AI, generative AI, large language models, multi-agent systems, systems biology

## Abstract

**Background:**

Cancer diagnostics is being reshaped by rapid advances in artificial intelligence, yet a persistent gap separates computational performance from clinical trust. Systematic reviews confirm that 83% of XAI studies in oncology excluded clinicians from development or evaluation, 87% lacked rigorous assessment of XAI explanations, and no universally accepted quality metrics for XAI outputs currently exist. Concurrently, generative AI (GenAI) is entering oncology at an unprecedented pace, yet it operates largely without formal interpretability governance.

**Aim:**

This review aimed to (i) synthesize the documented gaps in XAI applications for cancer diagnostics from peer-reviewed literature, (ii) evaluate the state and limitations of GenAI in oncological settings, and (iii) propose a conceptual framework of three GenAI-powered, XAI-governed agents designed to address these gaps within a systems biology context.

**Review Findings:**

A narrative synthesis of peer-reviewed literature published between 2020 and 2026 across PubMed, Scopus, and Web of Science identified four critical, recurrent gaps: systematic exclusion of clinicians from XAI development, the absence of standardized evaluation metrics, incomplete cross-omics explanations, and the near absence of lifestyle-driven XAI models for cancer risk. GenAI is accelerating in oncology but introduces additional safety concerns, including hallucinations, non-deterministic outputs, and the illusion of transparency through chain-of-thought reasoning.

**Framework Proposal:**

Three specialized agents are proposed: a Multi-Omics XAI Agent (MO-XAI Agent) for cross-layer biological data interpretation, a Clinician Trust and Communication Agent (CTC Agent) for structured explanation translation and quality scoring, and a Lifestyle-Driven Cancer Risk Stratification Agent (LRS Agent) for modifiable risk factor analysis. Each agent uses a large language model as the computational backbone with SHAP, LIME, or graph-level XAI methods as governance layers.

**Conclusion:**

To our knowledge, this framework represents the first conceptual architecture to integrate agentic GenAI with systematic XAI governance for cancer diagnostics, offering a clinically grounded, biologically interpretable, and regulatorily aligned design specification for future implementation research.

## 1 Introduction

Cancer remains the second leading cause of death worldwide, responsible for an estimated 9.7 million deaths in 2022 and approximately 20 million new diagnoses across all cancer types in the same year. Projections from the Global Cancer Observatory indicate a 77% increase in the annual case burden by 2050, driven primarily by demographic aging and the rising prevalence of modifiable risk factors, including obesity, physical inactivity, and tobacco use (Bray et al., 2024). Against this epidemiological background, artificial intelligence has emerged as a transformative force in oncological medicine, offering analytical capacities that exceed what is feasible through unaided clinical judgment. Machine learning models have demonstrated performance at or above a specialist level in skin lesion classification, radiological detection of pulmonary nodules, and pathological grading of prostate and colorectal tissue samples. Agentic AI systems, incorporating large language models (LLMs) and vision-language models, are now active across virtually every stage of the oncology workflow, from screening and diagnosis to prognosis and treatment planning (Rahman et al., 2025). A systematic review of 123 papers published between 2023 and 2025 confirmed that 91.9% of agentic AI studies in cancer detection appeared in 2024 and 2025, reflecting a rate of adoption with few precedents in the history of clinical computing (Rahman et al., 2025). The speed of this adoption, however, has not been matched by the development of the interpretability frameworks that safe clinical deployment requires. Clinicians making life-affecting diagnostic decisions need to understand not merely what a model predicts but why. Without that understanding, trust cannot be established, error cannot be identified, and accountability cannot be assigned. Opacity is not a secondary technical problem in oncological AI; it is the primary barrier to clinical adoption (Mohamed et al., 2025; Roustan and Bastardot, 2025).

Explainable artificial intelligence (XAI) was developed specifically to address model opacity. SHapley Additive exPlanations (SHAP), Local Interpretable Model-Agnostic Explanations (LIME), and Gradient-weighted Class Activation Mapping (Grad-CAM) are now standard tools in the XAI literature, applied across cancer subtypes, imaging modalities, and multi-omics datasets to surface the reasoning behind black-box predictions (Lundberg and Lee, 2017; Ribeiro et al., 2016; Abbas et al., 2025; Selvaraju et al., 2020). The field has grown substantially: XAI applications have been documented in breast, colorectal, lung, pancreatic, and cervical cancer, among others, and post hoc model-agnostic methods now constitute the majority of deployed XAI tools in clinical decision support systems (Abbas et al., 2025). The appeal of post hoc methods is practical: they require no architectural modification to the underlying model, can be applied to any trained classifier, and produce human-readable attribution scores. SHAP values in particular have found wide adoption because they are theoretically grounded in cooperative game theory and produce consistent, locally accurate feature attributions across diverse model types (Noor et al., 2025). In multi-omics contexts, XAI has been applied to link statistical cancer risk predictions to biological mechanisms and map model outputs to known pathway signatures (Zhang and Yin, 2025; Hsu et al., 2026). Applied to lifestyle and epidemiological data, SHAP has demonstrated utility in identifying the top modifiable contributors to cancer risk, offering a pathway toward individualized prevention guidance (Huang et al., 2025; Coupland et al., 2025). These applications represent genuine scientific contributions. Yet the translation of XAI research into clinical practice has been limited by structural failures that are now well documented across multiple systematic reviews and meta-analyses: clinicians are absent from the XAI development process in the overwhelming majority of studies, no standardized framework exists for evaluating explanation quality, single-modality data remains the norm despite the multi-level biological organization of cancer, and XAI applications for lifestyle-driven cancer risk remain at proof-of-concept stage (Mohamed et al., 2025; Noor et al., 2025; Brankovic et al., 2025; Shifa et al., 2025).

The entry of generative AI into this landscape adds both capability and complexity. LLMs can generate structured diagnostic reports, assist with multi-disciplinary tumor board decisions, process heterogeneous clinical text, and now serve as reasoning engines within multi-agent diagnostic architectures (Iannantuono et al., 2023; Carl et al., 2024; Yum et al., 2025). Generative models, including generative adversarial networks (GANs) and diffusion models, produce synthetic medical images for dataset augmentation, addressing one of the most persistent barriers to AI development in oncology: data scarcity in rare cancer types (Koetzier et al., 2024; Yum et al., 2025; Wang et al., 2025; Barki et al., 2024). These capabilities are real and clinically significant. The safety risks, however, are equally real. Hallucination, in which LLMs generate confident but factually incorrect outputs, has been documented in clinical contexts with direct implications for patient safety (Roustan and Bastardot, 2025; Bélisle-Pipon, 2024). Chain-of-thought reasoning, often presented as evidence that LLMs are interpretable, produces narrative descriptions of apparent reasoning without exposing the actual generative process (Derbal, 2025). Multi-domain empirical evaluations of LLM performance in health contexts, including assessments in mental health (Dergaa et al., 2024a), personalized nutrition guidance (Dergaa et al., 2024b), exercise prescription (Dergaa et al., 2024c), and medical writing (Dergaa et al., 2024d), consistently identify the same pattern: strong performance on bounded, well-defined tasks alongside critical failures in scenarios requiring nuanced contextual clinical reasoning. Fairness presents a further challenge: LLM-based oncological tools exhibit racial and gender biases that could widen, rather than narrow, existing cancer care disparities (Agrawal, 2024; Ktena et al., 2024). The field, therefore, faces a paradox: the most powerful AI tools for cancer diagnostics are also the least interpretable, and the XAI infrastructure that would govern their clinical deployment does not yet exist at an adequate level.

Based on these identified gaps, the present review aimed to address this paradox directly. Three specific objectives were pursued: (i) to systematically map the documented failures in XAI for cancer diagnostics as established in the current peer-reviewed literature; (ii) to evaluate the state, capabilities, and interpretability limitations of GenAI in oncological applications; and (iii) to propose a conceptual agentic framework consisting of three GenAI-powered, XAI-governed agents that directly address each documented gap within a systems biology framing. The framework builds on prior clinical AI infrastructure developed by the research group, including a hybrid deep learning approach for cancer pathology text processing using BioClinicalBERT enhanced by retrieval-augmented generation (Abdaoui et al., 2025; Barki et al., 2021), a structured AI integration framework for critical clinical settings (Boussi Rahmouni et al., 2025), and multi-domain empirical evaluations of LLM performance across health contexts (Dergaa et al., 2024a; Dergaa et al., 2024b; Dergaa et al., 2024c; Dergaa et al., 2024d; Dergaa et al., 2023; Dergaa et al., 2024e; Washif et al., 2024; Methnani et al., 2023). Importantly, the proposed framework is not intended to replace established clinical decision pathways. Rather, all diagnostic recommendations generated by the agentic architecture are designed to operate within the boundaries of evidence-based oncology practice, including internationally recognized clinical guidelines such as those developed by the National Comprehensive Cancer Network (NCCN) and the European Society for Medical Oncology (ESMO) (Ong et al., 2026; Armoundas and Singh, 2026). These guidelines provide the clinical context necessary for selecting, sequencing, and interpreting diagnostic investigations, while patient-specific comorbidities and clinical constraints remain essential components of expert review and multidisciplinary decision-making. Accordingly, the proposed framework should be viewed as a decision-support infrastructure that augments, rather than substitutes for, guideline-driven clinical judgment.

## 2 XAI in cancer diagnostics: current evidence and documented gaps

This study adopts a structured narrative review approach aimed at synthesizing current evidence on XAI in cancer diagnostics and identifying documented methodological and clinical gaps. The review was designed to provide a comprehensive conceptual mapping rather than a systematic meta-analysis. A literature search was conducted using major scientific databases, including PubMed, Scopus, IEEE Xplore, and Web of Science, covering publications primarily between 2020 and 2026, reflecting the rapid expansion of XAI in oncology. Keywords included combinations of “explainable AI,” “XAI,” “cancer diagnostics,” “machine learning interpretability,” “SHAP,” “lime,” “Grad-CAM,” and “multi-omics cancer AI.” Studies were included if they (i) focused on AI applications in cancer diagnosis, prognosis, or treatment decision support, and (ii) incorporated or discussed explainability or interpretability methods. Studies were excluded if they were not related to oncology, did not involve AI-based methods, or focused exclusively on non-explainable predictive models without interpretability analysis. Data extraction focused on four analytical dimensions aligned with the structure of this review: (1) XAI methods and modalities, (2) clinical application domain, (3) evaluation strategies for interpretability, and (4) reported limitations or gaps. Extracted findings were synthesized using a thematic analysis approach, enabling identification of recurring structural gaps across clinician involvement, standardization, multi-omics integration, and lifestyle-based modeling. Given the heterogeneity of methodologies and evaluation metrics across included studies, a qualitative synthesis was prioritized over a quantitative meta-analysis.

### 2.1 The state of the field

XAI applications in oncology have expanded across nearly every major cancer type and data modality since 2020. In imaging, Grad-CAM and attention mechanisms dominate; in tabular and omics data, SHAP and lime are the methods of choice; in high-dimensional transcriptomic survival analysis, SurvSHAP and DeepSHAP have emerged as domain-specific variants with favorable stability and biological consistency properties (Zuo et al., 2026). A meta-analysis of 62 studies across clinical decision support systems confirmed that model-agnostic *post hoc* techniques now constitute the majority of XAI tools deployed in oncology, reflecting a field preference for methods that require no modification to trained model architectures (Abbas et al., 2025). Reviews of XAI in electronic health record-based oncology document a similar picture: SHAP, permutation importance, and partial dependence plots dominate, with applications spanning gastric, colorectal, lung, and breast cancer risk prediction from structured EHR data (Yang and Liu, 2026). The adoption of *post hoc* XAI carries a fundamental limitation, however. These methods explain a model’s output without guaranteeing that the explanation reflects the model’s internal reasoning; their fidelity is sensitive to input perturbations and simplifying assumptions that may invalidate the attribution in non-standard clinical presentations (Mohamed et al., 2025).

**FIGURE 1:**
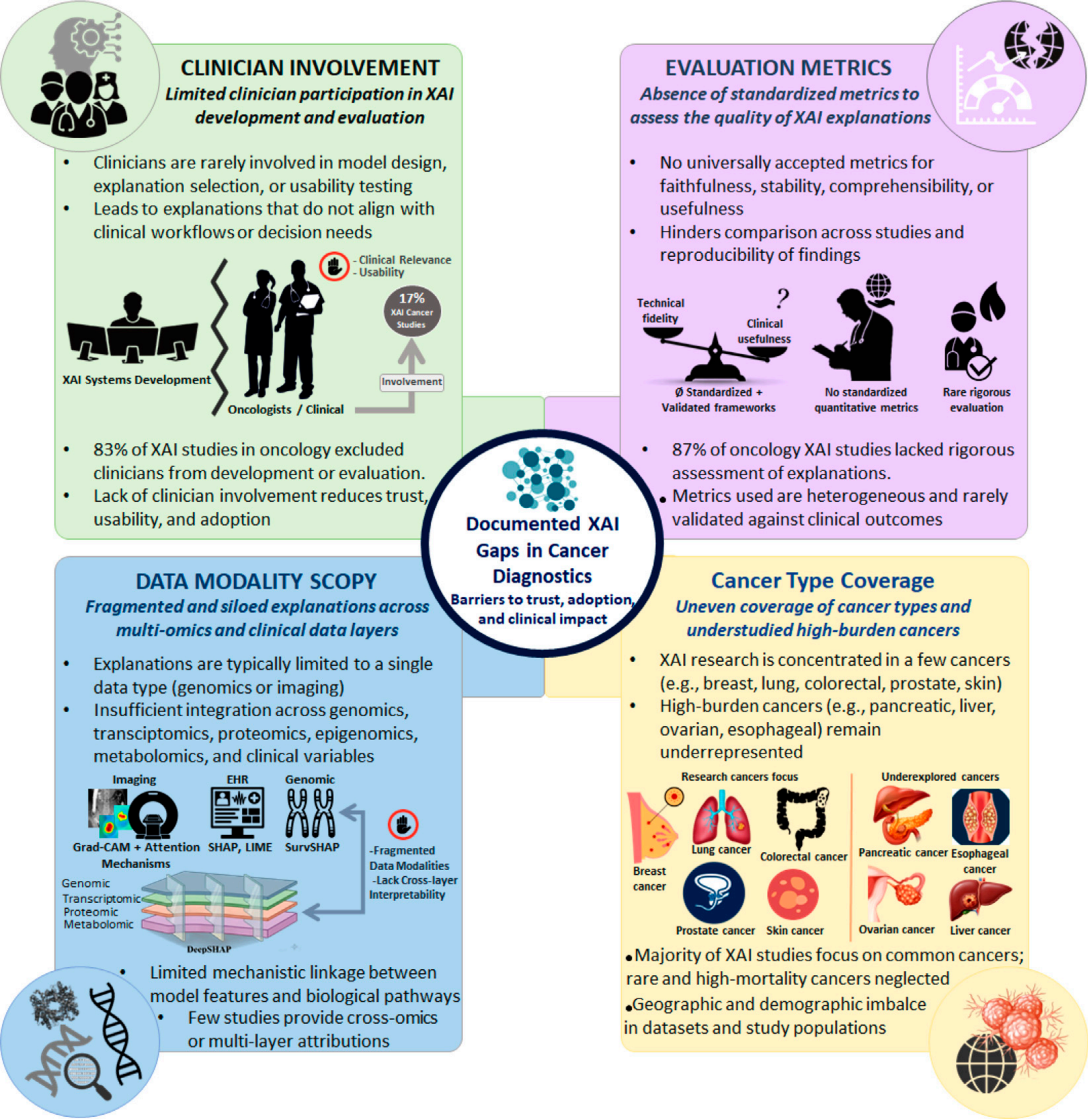
Conceptual map of documented XAI gaps in cancer diagnostics | Evidence-based gap taxonomy across four dimensions.

Reviews focused on specific cancer-modality pairings document a consistent pattern of superficiality. The most thorough analysis of XAI in mammography for breast cancer screening found that existing solutions focus on lesion localization and saliency maps rather than actionable clinical insights, and identified the lack of standardized quantitative metrics for explanation quality as the most critical methodological gap in the field (Shifa et al., 2025). In pancreatic cancer, among the most lethal malignancies with a 5-year survival rate below 12%, a review of 21 machine learning studies found that only three explicitly integrated any XAI method (Alharbi and Alfayez, 2025). This gap is striking in precisely the clinical context where early AI-assisted detection could have the greatest life-saving impact. Figure 1 summarizes the major documented gaps in explainable artificial intelligence (XAI) for cancer diagnostics, organized across four dimensions: clinician involvement, evaluation metrics, data modality scope, and cancer type coverage.

### 2.2 The clinician exclusion problem

The most consequential structural failure in the XAI-oncology literature is not technical but organizational: clinicians are systematically absent from the development and evaluation of the tools intended to support their decisions. The landmark systematic review by Mohamed and colleagues, which analyzed 69 studies covering diagnosis, prognosis, and treatment planning, found that clinicians were excluded from 83% of XAI cancer studies and that 73% excluded clinicians specifically from system design (Mohamed et al., 2025). The same review found that 87% of included studies lacked rigorous assessment of XAI explanations, indicating that technical development consistently outpaced quality evaluation. A complementary meta-analysis of 62 clinical decision support studies found no research that evaluated explanation fidelity, clinician trust, or usability in real-world settings (Abbas et al., 2025). Across the mammography XAI literature, studies consistently report that rigorous explainability remains an unmet priority, with existing solutions stopping short of prescribing standardized methods for clinical evaluation (Shifa et al., 2025). The consequence is a field producing tools optimized for computational benchmarks on validation datasets rather than for the reasoning needs of an oncologist reviewing a scan under time pressure in a clinical setting. Explanations that are technically faithful to a model’s predictions may be clinically useless, confusing, or harmful if they present information in formats that do not match clinical decision-making patterns.

### 2.3 The standardization deficit

The absence of standardized metrics for evaluating XAI outputs in clinical settings is the second major structural gap. No validated instrument currently exists that captures both the technical fidelity of an explanation and its clinical relevance. A significant research effort recently produced the Clinician-informed XAI Evaluation Checklist with Metrics (CLIX-M), a 14-item instrument covering clinical, machine-learning, and decision attributes (Brankovic et al., 2025). Its authors explicitly described it as a first step toward standardization, emphasizing that it requires prospective validation in clinical settings before adoption as a reporting standard (Brankovic et al., 2025). The WIREs review of XAI in healthcare, covering 89 publications across 19 medical domains, confirmed that the lack of standardized evaluation metrics and practical obstacles in integrating XAI systems into clinical workflows are the two most consistently emphasized research gaps in the field (Noor et al., 2025). This evaluation deficit is not a marginal concern: without agreed metrics, the XAI literature cannot accumulate comparative knowledge, clinicians cannot assess whether one explanation approach is better than another for their specific context, and regulators cannot establish minimum transparency standards for AI-assisted cancer diagnostics.

### 2.4 Multi-omics and cross-layer gaps

Cancer biology is inherently multi-level. Tumors arise from and are sustained by the coordinated disruption of genomic, transcriptomic, proteomic, and metabolic processes. Integrated multi-omics classifiers consistently outperform single-modality models, with recent analyses reporting AUCs of 0.81–0.87 for early cancer detection using cross-omics feature integration (Hsu et al., 2026). The systems biology imperative, integrating signals across biological layers to understand cancer as a network phenotype, is well established at the conceptual level. Its translation into XAI practice has been incomplete. Most XAI systems in multi-omics oncology apply a single explanation method to the model’s final output without distinguishing the explanatory contribution of each biological stratum (Zhang and Yin, 2025). Cross-layer interpretability, which would reveal not merely which feature drove a prediction but which biological pathway and which omics layer that feature belongs to, remains largely absent from the published literature. A pan-cancer benchmarking study across 15 cancer types found that while DeepSHAP performed well for identifying prognostic biomarkers in transcriptomic survival models, explanation stability across cancer types remained a major unresolved challenge (Zuo et al., 2026). For immunologists and systems biologists, XAI output that cannot be mapped to tumor microenvironment interactions or pathway signatures offers little scientific or clinical value beyond the original prediction score. These limitations are not unique to molecular data and extend to other domains of cancer risk modeling, suggesting a broader gap in the alignment between predictive explanations and clinically actionable interpretation.

### 2.5 The lifestyle-driven XAI gap

A parallel gap exists for lifestyle-driven cancer risk stratification. Physical inactivity, dietary patterns, excess adiposity, and metabolic dysregulation are established drivers of colorectal, breast, and endometrial cancer risk (Huang et al., 2025). These risk factors are modifiable, making XAI for lifestyle-based cancer risk prediction a direct instrument of personalized cancer prevention. SHAP applied to lifestyle and longitudinal epidemiological data has demonstrated predictive utility, but has been found to misalign with causal relationships in ways that limit actionability (Coupland et al., 2025). Specifically, high SHAP attribution for a feature in a predictive model does not imply that modifying that feature will reduce cancer risk, because SHAP captures statistical association and not causal effect. In life course data, where risk factors are temporally ordered and mutually confounded, this distinction is clinically critical: a recommendation to modify a high-SHAP predictor that is actually a proxy for an unmodifiable confounder is not only unhelpful but potentially misleading (Coupland et al., 2025). The XAI literature in this space has not yet addressed this causal interpretation problem systematically. This limitation mirrors the challenges observed in multi-omics XAI systems, where feature attribution fails to reflect underlying causal or mechanistic relationships across biological layers.

Taken together, both multi-omics and lifestyle-driven models highlight a shared interpretability gap: current XAI methods primarily quantify statistical contribution rather than causal relevance, limiting their ability to connect predictive signals across biological and behavioral scales into a unified and clinically actionable framework.

## 3 Generative AI in oncology: acceleration, capability, and interpretability deficit

### 3.1 The scale and speed of GenAI adoption

The integration of generative AI into oncological workflows represents one of the fastest technology adoption trajectories in modern medicine. The transition from proof-of-concept demonstrations to active clinical testing occurred within approximately 2 years of the release of powerful multimodal LLMs. Applications now span the complete oncology workflow. LLMs generate and check radiology and pathology reports (Iannantuono et al., 2023; Yang et al., 2025), assist with tumor board documentation (Yum et al., 2025), support clinical decision-making in lung, colorectal, and breast cancer (Carl et al., 2024), and are being evaluated in multi-agent frameworks for multidisciplinary consultation (Rahman et al.; Chen et al., 2025). In imaging, generative models, including GANs and diffusion models, produce synthetic medical images for dataset augmentation, modality translation, and rare pathology simulation (Koetzier et al., 2024; Wang et al., 2025). Recent discussions in the literature have highlighted the growing interest in generative AI for cancer detection and diagnosis, including potential applications such as AI-generated digital twins for personalized cancer screening (Singh et al., 2024). However, these perspectives should be interpreted cautiously, as they are often based on conceptual or opinion-driven discussions rather than validated clinical evidence (Singh et al., 2024). Large language model performance benchmarks have reinforced the optimism: GPT-4 versions matched pathologist-level error detection in pathology reports (89.5% vs. 88.5%), equaled dermatologist performance in skin lesion classification (84.8% vs. 84.6%), and achieved 97% accuracy in ovarian cancer staging against 88% by radiologists (Rahman et al., 2025).

### 3.2 Interpretability limitations of generative AI

While the performance record of LLMs is well established, their structural limitations must also be acknowledged. These models are generative systems that produce outputs by sampling from learned probability distributions over token sequences. Although chain-of-thought reasoning is often interpreted as evidence of interpretability, it primarily generates plausible narrative explanations rather than exposing the actual internal computational process (Derbal, 2025). The distinction matters enormously in clinical oncology. A clinician reading a chain-of-thought justification for an AI-generated cancer diagnosis recommendation cannot determine whether the narrative accurately describes the model’s reasoning or is a post hoc rationalization generated after the prediction. Applying XAI methods such as post hoc attribution techniques to highlight the input features most strongly associated with the output represents a more reliable approach to surfacing what drove a generative model’s prediction (Derbal, 2025). Hallucination introduces an additional safety dimension that is particularly critical in clinical settings. Confident generation of factually incorrect medical information has been widely documented across medical domains. Clinical safety analyses show that hallucinated content can vary in severity, ranging from minor factual inconsistencies to unsupported treatment recommendations that may directly harm patients (Roustan and Bastardot, 2025; Bélisle-Pipon, 2024). Roustan and Bastardot categorized hallucination risks and proposed institutional-scale governance requirements before LLM deployment in high-stakes clinical environments (Roustan and Bastardot, 2025).

Bias can be considered as a third dimension of risk in clinical applications of LLMs. Indeed, LLMs and vision-language models trained on datasets that underrepresent specific demographic groups have been shown to exhibit racial and gender biases in oncological recommendations (Agrawal, 2024). Bias recognition and mitigation must be addressed across the complete AI model lifecycle, from training data curation through deployment and longitudinal surveillance (Hasanzadeh et al., 2025). Generative models have shown promise in partially mitigating these disparities by generating synthetic data for underrepresented subgroups, improving classifier fairness under distribution shift (Ktena et al., 2024; Marchesi et al., 2025). But synthetic data generation is not a complete solution: models trained on biased corpora will generate biased synthetic data, and demographic overrepresentation of high-income Western populations in epidemiological training datasets requires structural remediation, not generation-based workarounds.

### 3.3 The case for XAI governance of GenAI

The convergence of these findings produces a clear design requirement. Generative AI in oncological diagnostics requires XAI governance built into the system architecture, not appended as a post hoc audit. Multi-domain empirical evaluations of LLM performance in health contexts confirm that the pattern of capability combined with interpretability failure is not restricted to oncology. Evaluations of ChatGPT in mental health assessment found it inadequate for complex clinical scenarios requiring contextual judgment (Dergaa et al., 2024a). Critical assessments of GPT-4 in personalized health promotion found that clinical reasoning gaps necessitate professional oversight in all applications involving tailored recommendations (Dergaa et al., 2024b; Dergaa et al., 2024c). Analysis of ChatGPT in medical writing identified that its limitations are most consequential precisely where clinical stakes are highest, in complex, multi-factor diagnostic decisions (Dergaa et al., 2024d). The research team’s prior experience generating natural language clinical summaries from cancer pathology reports using BioClinicalBERT enhanced with RAG demonstrates both the feasibility and the requirements of GenAI applied to oncological text (Abdaoui et al., 2025): clinical grounding through retrieval augmentation, domain-specific fine-tuning, and structured output validation are all necessary for reliable performance. These requirements point directly toward the governance architecture that the proposed framework operationalizes.

## 4 A proposed agentic XAI framework for cancer diagnostics

### 4.1 Framework architecture and systems biology rationale

The proposed framework consists of three modular, task-specific agents. Each agent is designed to address one or more of the documented gaps identified in Section 2. All three share a common architectural logic: a large language model, optionally augmented with domain-specific fine-tuning and retrieval augmentation, serves as the computational backbone, while a named XAI method, such as SHAP, lime, or GNNExplainer, serves as the governance layer producing auditable, human-interpretable explanations. The agents can operate independently for single-domain diagnostic queries or in coordinated deployment for complex multi-modal assessments. An orchestration layer serves as the coordination and control mechanism of the framework. It receives external diagnostic inputs, determines which specialized agents should be activated according to the diagnostic context, manages inter-agent communication and execution sequencing, and collects their outputs. The agents themselves do not generate final clinical reports; instead, each produces intermediate analytical outputs such as pathway rankings, explanation quality scores, diagnostic confidence measures, and cancer probability estimates. After all required agents complete execution, the Orchestration Layer performs a structured aggregation and alignment process that integrates these outputs and synthesizes them into a single Unified Clinical Report presented in natural language. The execution workflow proceeds as follows: (1) diagnostic inputs are received by the Orchestration Layer; (2) selected agents are executed either independently or sequentially depending on data dependencies and diagnostic complexity; (3) intermediate outputs are exchanged and consolidated; and (4) the Orchestration Layer generates the final Unified Clinical Report. The orchestration component does not perform domain-level analysis itself and does not permanently store input data; its role is limited to workflow coordination, output integration, and report generation.

The framework is grounded in a systems biology perspective in two senses. At the biological level, the diagnostic data it processes reflects the multi-level organization of cancer: from molecular pathway disruptions captured by omics profiles, to tissue-level features captured by imaging, to organism-level behavioral and metabolic signals captured by lifestyle variables (Heo et al., 2021). At the clinical level, the framework is designed to support, rather than replace, evidence-based oncology practice. Before any diagnostic recommendation is forwarded to clinicians, outputs are evaluated in the context of disease-specific oncology guidelines (e.g., NCCN, ESMO), patient-specific clinical characteristics, and relevant comorbid conditions. Any discrepancy, uncertainty, or guideline inconsistency triggers human expert review and multidisciplinary validation. At the sociotechnical level, the framework treats the clinician-AI diagnostic interaction as a system in which computational tools, institutional workflows, regulatory requirements, and human reasoning are mutually constitutive (Salwei and Carayon, 2022). Tools designed to optimize computational metrics without attending to clinical, regulatory, and behavioral dimensions reproduce the same failures that have limited XAI adoption to date (Mohamed et al., 2025; Noor et al., 2025; Brankovic et al., 2025). The framework’s design addresses all three dimensions explicitly.

**FIGURE 2:**
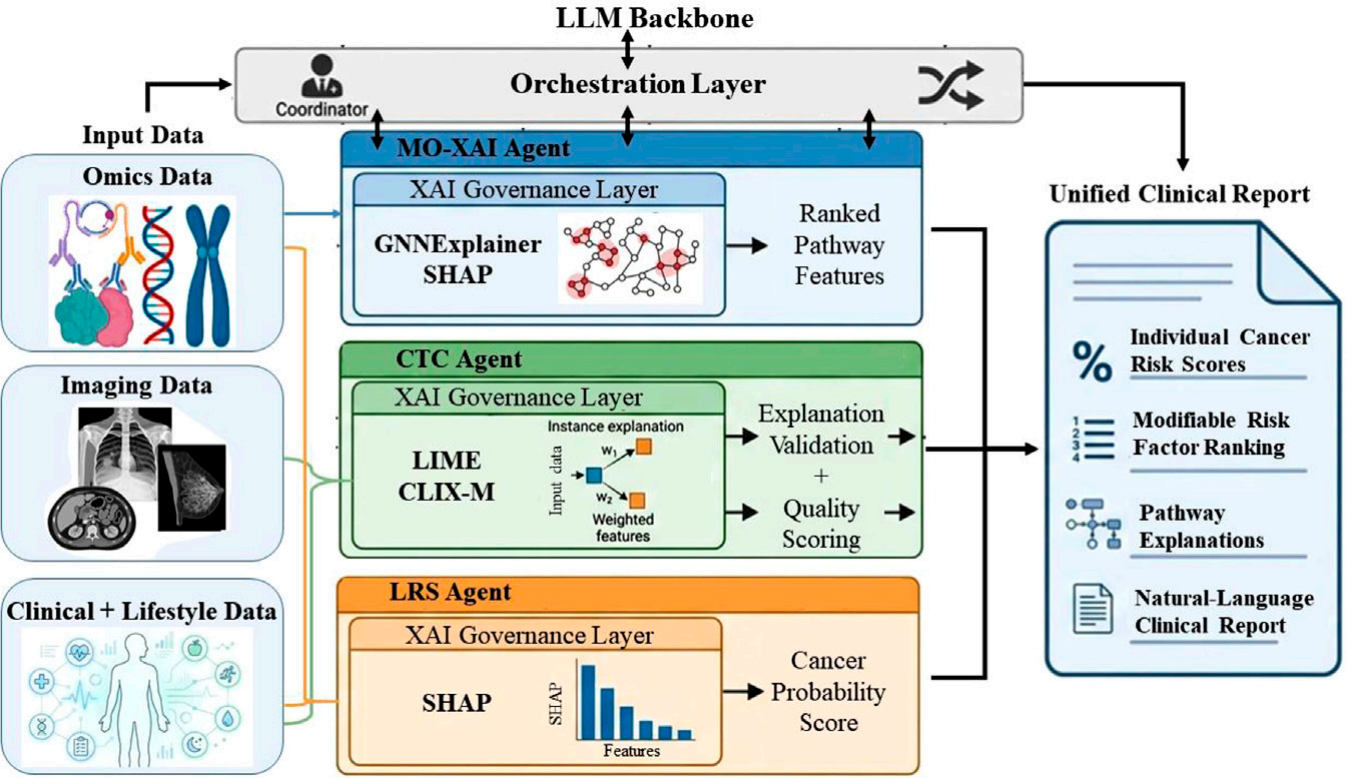
Overview of the architecture of the three-agent Agentic XAI Framework with orchestration layer and clinical output pathways.

This architecture extends the Healthcare 5.0 Learn-Predict-Monitor-Detect-Correct framework developed by the research team, which provides structured governance stages for AI integration into critical clinical settings (Boussi Rahmouni et al., 2025). The three agents correspond to the Predict, Detect, and Monitor functions of that framework, applied specifically to the cancer diagnostics context. Data governance and privacy compliance draw on blockchain-based HIPAA and GDPR frameworks developed and validated by the same team (Barbaria et al., 2025). Figure 2 provides an overview of the proposed Agentic XAI Framework, including the three specialized agents, the orchestration layer, the shared LLM backbone, the XAI governance mechanisms, and the unified clinical reporting workflow.

### 4.2 Agent 1: the multi-omics XAI agent (MO-XAI agent)

The MO-XAI Agent addresses the cross-layer interpretability gap in multi-omics cancer analytics. Standard XAI applications treat multi-omics data as a flat feature space, applying a single explanation method to the model’s final prediction without distinguishing contributions by biological stratum. This design choice produces SHAP values or attribution scores without the layer-level attribution that would allow a clinician or molecular biologist to identify which pathway, and from which omics source, most strongly drove the risk prediction. The MO-XAI Agent processes multi-layered omics data through a modular pipeline in which each omics stratum is handled by a dedicated sub-model.

For tabular omics data, including gene expression matrices, metabolite concentration profiles, and protein abundance arrays, SHAP values are computed at the layer level independently before cross-layer aggregation. For network-level data derived from protein-protein interaction or gene regulatory networks, a Graph Neural Network architecture is paired with GNNExplainer to identify the network subgraphs whose activation most strongly influences the cancer risk prediction (Zhang and Yin, 2025; Zuo et al., 2026; Ying et al., 2019). An LLM with domain-specific fine-tuning on biological pathway databases, including KEGG, Reactome, and GO, serves as the orchestration and interpretation engine. It receives the layer-level SHAP values and graph-level subgraph attributions as structured input and generates a natural-language summary mapping the prediction to known biological mechanisms.

**FIGURE 3:**
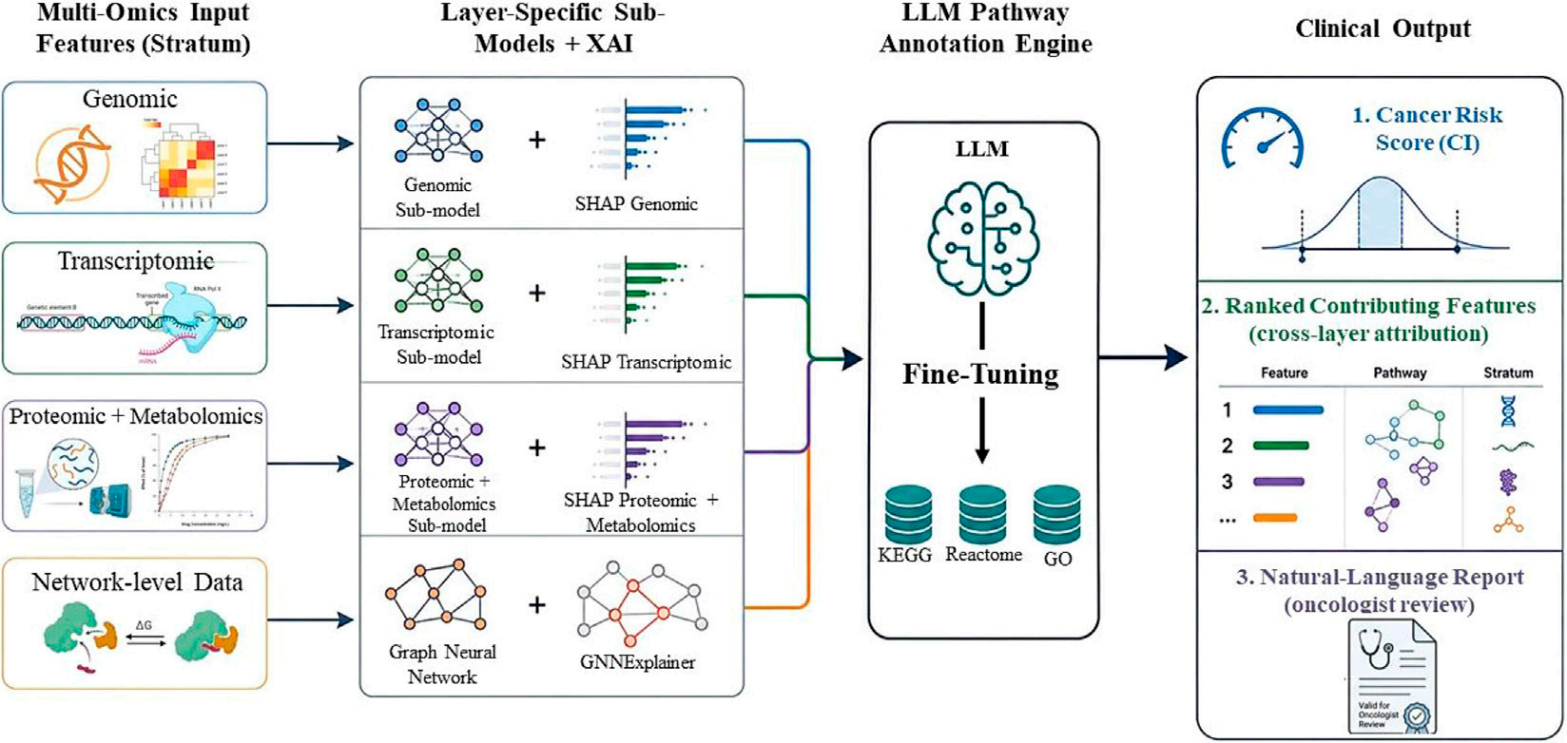
Conceptual overview of the MO-XAI Agent architecture: multi-omics integration, cross-layer SHAP attribution, and LLM-assisted pathway interpretation.

The clinical output of the MO-XAI Agent includes three components: a cancer risk score with associated confidence interval; a ranked list of contributing biological features with cross-layer attribution specifying pathway membership and biological stratum; and a natural-language report suitable for oncologist review. Public datasets from The Cancer Genome Atlas provide a near-term validation pathway given their comprehensive genomic, transcriptomic, and clinical variable coverage across 33 cancer types (Hsu et al., 2026). The TCGA pan-cancer cohort is particularly well suited to Agent 1 validation because it allows cross-cancer generalizability testing, addressing the single-cancer-type limitation that characterizes most current XAI oncology research (Zuo et al., 2026; Yang and Liu, 2026). Figure 3 illustrates the architecture and workflow of the Multi-Omics XAI Agent (MO-XAI Agent), highlighting multi-omics integration, layer-specific explainability, pathway interpretation, and clinically interpretable outputs.

### 4.3 Agent 2: the clinician trust and communication agent (CTC agent)

The CTC Agent addresses the clinician exclusion and standardization gaps simultaneously and does so through a design principle that distinguishes it from existing XAI approaches: it does not build a new predictive model. It functions as an explanation, translation, and quality governance layer that sits downstream of any XAI-equipped cancer AI system, receiving raw explanation outputs and converting them into clinician-readable, quality-evaluated reports. This separation of concerns, standard in software systems engineering but rare in clinical AI design, allows the CTC Agent to be applied to existing cancer AI deployments without requiring architectural modification to upstream systems.

The agent’s processing pipeline operates across three stages. In the first stage, a multimodal LLM receives the raw XAI output generated by an upstream cancer AI system, which may include a SHAP value vector, a Grad-CAM saliency map, or a lime feature list. These inputs are accompanied by contextual clinical variables that are assumed to be already available from prior diagnostic assessment, including confirmed or suspected cancer type, clinical stage, comorbidities, and treatment history. These contextual variables are not used to establish a new diagnosis; rather, they provide clinical grounding for adapting and translating the explanation into a form that is meaningful, actionable, and appropriate for clinician interpretation. The LLM generates a candidate explanation in a structured schema comprising six components: (i) the predicted outcome with its confidence level; (ii) the most influential features with biological interpretation; (iii) features identified as non-contributory and the rationale for their exclusion; (iv) limitations and uncertainty associated with the prediction; (v) a context-aware recommended clinical follow-up action; and (vi) the evidential quality supporting that recommendation. Importantly, the recommended clinical action is not intended to establish a diagnosis or prescribe treatment. Instead, it provides a clinically relevant interpretation of the AI prediction, such as suggesting additional diagnostic investigations, closer monitoring, specialist referral, or multidisciplinary review, thereby enhancing the actionability of the explanation while maintaining clinician responsibility for all diagnostic and therapeutic decisions. In the second stage, the candidate explanation is scored against a clinical quality rubric adapted from the CLIX-M framework (Brankovic et al., 2025), assessing faithfulness, completeness, actionability, and communicative clarity. Explanations falling below defined thresholds on any dimension are flagged for human expert review before clinical delivery. In the third stage, the validated explanation is formatted for the relevant clinical context: a summary for time-constrained screening workflows, an extended annotated report for tumor board deliberations, or a patient-facing version for shared decision-making consultations.

**FIGURE 4:**
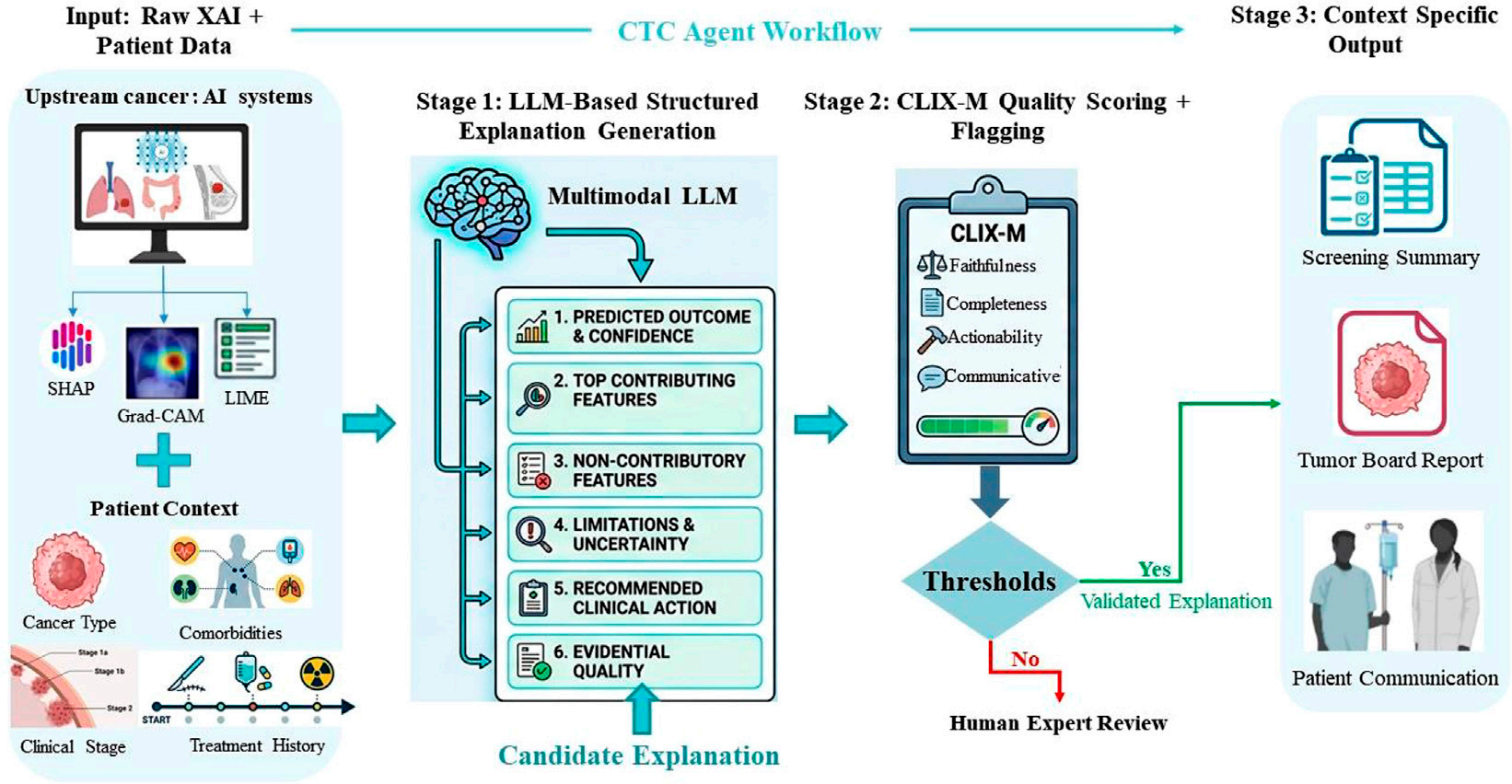
CTC Agent: explanation translation, quality scoring, and context-specific clinical formatting workflow.

The CTC Agent’s role as an explanation translation layer has a direct precedent in the research team’s BioClinicalBERT work, which demonstrates that GenAI can process oncological pathology text in ways that are both clinically grounded and technically auditable (Abdaoui et al., 2025). The CTC Agent extends this capability from text processing to explanation governance, applying the same principle of structured, quality-evaluated output to any XAI system in the oncology pipeline. Its implementation directly instantiates the CLIX-M checklist as an operational standard rather than an aspirational framework, providing the first proposal in the literature for building XAI quality standards into the AI pipeline as an architectural requirement rather than a *post hoc* audit step (Brankovic et al., 2025). Figure 4 illustrates the downstream workflow of the Clinician Trust and Communication Agent (CTC Agent). The figure emphasizes the transformation of raw XAI outputs into structured, quality-scored, and context-adapted explanations using pre-existing patient context information rather than performing additional diagnostic inference.

### 4.4 Agent 3: the lifestyle-driven cancer risk stratification agent (LRS agent)

The LRS Agent addresses the lifestyle-driven XAI gap and represents the framework’s most direct contribution to cancer prevention and public health. Cancers with established lifestyle etiology, primarily colorectal, breast, and endometrial cancer, account for a substantial proportion of preventable cancer cases globally. Lifestyle risk factor modification is the most scalable cancer prevention strategy available, yet the XAI tools that would translate modifiable risk factor predictions into actionable individual guidance remain underdeveloped (Huang et al., 2025; Coupland et al., 2025). The LRS Agent is designed to fill this space.

The agent processes a curated panel of lifestyle and metabolic variables drawn from validated epidemiological instruments: objective or validated self-report physical activity; dietary pattern indices; anthropometric measures including body mass index and waist-to-hip ratio; inflammatory biomarkers including C-reactive protein and interleukin-6; hormonal markers including insulin, estradiol, and insulin-like growth factor 1; and self-reported behavioral risk factors including smoking status, alcohol consumption, and sedentary time. The prediction model uses an XGBoost ensemble architecture for its established performance in tabular health data and its compatibility with SHAP computation (Huang et al., 2025). SHAP values are computed at both individual and population levels, producing two output types: an individual risk report ranking modifiable risk factors by their contribution to that patient’s cancer risk score, and a population-level summary identifying the lifestyle variables most strongly associated with cancer risk stratification across the validation dataset.

**FIGURE 5:**
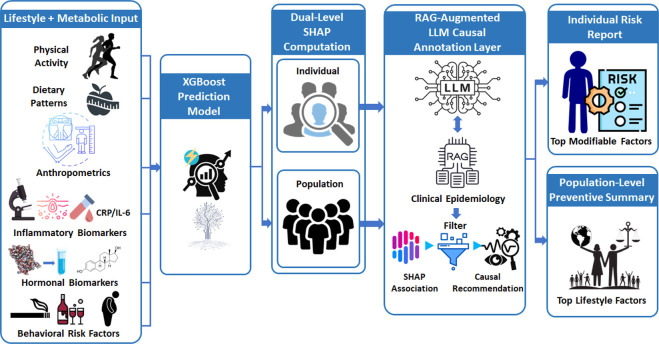
LRS Agent: lifestyle-driven cancer risk stratification with causal-annotated SHAP explanations via RAG-augmented LLM.

The LRS Agent’s integration with its LLM backbone addresses the critical causal interpretation problem identified in SHAP applications to life course data (Coupland et al., 2025). High SHAP attribution for a lifestyle feature does not imply that modifying that feature will reduce cancer risk; SHAP captures statistical association, not causal effect. In epidemiological data where risk factors are temporally ordered and mutually confounded, SHAP-based recommendations taken at face value may be incorrect or misleading. The LLM component, grounded in clinical epidemiology databases through RAG, provides causal annotation for each SHAP-ranked factor, distinguishing established causal risk factors from confounded associations and calibrating recommendation strength accordingly (Neha et al., 2025). This design directly addresses the causal misalignment limitation documented by Coupland and colleagues (Coupland et al., 2025). Target cancer types for initial validation include colorectal, breast, and endometrial cancer, given the robustness of their lifestyle risk factor evidence base and the availability of large-scale epidemiological datasets, including the UK Biobank and the Women’s Health Initiative. Figure 5 illustrates the Lifestyle-Driven Cancer Risk Stratification Agent (LRS Agent), including lifestyle and metabolic data inputs, SHAP-based risk attribution, RAG-supported causal interpretation, and prevention-oriented clinical outputs.

### 4.5 Orchestration and inter-agent coordination

The three agents share a data communication interface and are coordinated through a lightweight LLM-based orchestration layer. The orchestration layer receives clinical queries in natural language, determines which agents are relevant given the available data and clinical context, sequences their activation, and manages any data sharing between agents. Beyond coordinating the execution of AI agents, the orchestration layer serves as a workflow-awareness component that evaluates the completeness and sequencing of the available diagnostic information. Using disease-specific diagnostic pathways and institutional workflow rules, the layer can identify missing investigations, incomplete data elements, or inconsistencies between recommended and completed diagnostic procedures. When such gaps are detected, the system generates workflow alerts and recommendations for clinician review, thereby supporting multidisciplinary coordination and reducing the risk of incomplete diagnostic evaluation. Final responsibility for scheduling, patient communication, and clinical decision-making remains with healthcare professionals. For a patient presenting with both a suspicious genomic panel and a high lifestyle risk profile, the MO-XAI Agent and LRS Agent are activated in parallel, and their outputs are passed simultaneously to the CTC Agent for integrated explanation generation. For a screening-level query involving only lifestyle and metabolic data, only the LRS Agent and CTC Agent are activated, maintaining computational efficiency.

The orchestration architecture follows the multi-agent coordination principles documented in clinical AI literature, where a multi-agent conversational framework significantly outperformed monolithic single-agent models, improving the accuracy of the most likely diagnosis from 19.65% to 34.11% during primary consultations when using GPT-4 as the base model (Chen et al., 2025). The addition of XAI governance at each agent node, rather than only at the system’s final output, is the design innovation that distinguishes this framework from existing multi-agent clinical AI architectures (Salehi et al., 2025). Each agent is required to produce an auditable, quality-scored explanation before passing its output to the orchestration layer. This design prevents the common failure mode in which explanations are generated only for the aggregate system output, without traceable attribution to individual analytical decisions.

## 5 Implementation, data sources, and regulatory considerations

### 5.1 Data sources and validation pathways

Validation of the proposed framework requires datasets combining cancer outcomes with multi-omics profiles, lifestyle variables, and longitudinal follow-up. The Cancer Genome Atlas provides the most accessible resource for MO-XAI Agent validation, with comprehensive genomic and transcriptomic data for 33 cancer types linked to clinical outcomes (Hsu et al., 2026). The UK Biobank, with approximately 500,000 participants, linked genomic data, multi-modal imaging, lifestyle questionnaires, accelerometry, and incident cancer diagnoses, provides the most suitable resource for LRS Agent validation. For the CTC Agent, validation requires prospective collection of clinician assessments of explanation quality paired with diagnostic decision records and patient outcome data; no standardized dataset of this type currently exists, underscoring the urgency of the research infrastructure investment embedded in the agent’s design.

Electronic health record-derived datasets from clinical institutional partners extend validation to real-world conditions, capturing the data quality limitations, missing data patterns, and workflow constraints that academic cohort datasets do not reflect. Federated learning architectures allow multi-site validation without requiring centralized aggregation of protected health information, preserving patient privacy while enabling the sample diversity needed to assess cross-population generalizability (Barbaria et al., 2025). The thyroid cancer incidence data from Northern Tunisia generated by the research team (Khiari et al., 2026), combined with the regional epidemiological context it provides, indicates the importance of validation in non-Western populations where cancer risk factor profiles and healthcare system characteristics may differ substantially from the populations that dominate existing XAI oncology research.

### 5.2 Regulatory alignment

The proposed framework was designed with regulatory requirements as structural constraints. The EU AI Act, which entered into force in August 2024, classifies AI systems used in cancer diagnostic or treatment support as high-risk applications, triggering requirements for conformity assessment, transparency reporting, bias monitoring, and human oversight provisions (Ong et al., 2026). The FDA has adopted a cautious stance toward generative AI in regulated clinical settings, emphasizing validation requirements, predetermined change control plans, and continuous post-market performance monitoring (Ong et al., 2026; Armoundas and Singh, 2026). These requirements represent the minimum governance expectations that clinical oncological AI systems will face in major markets; the framework proposed here addresses them by design rather than by retrofit.

Each agent’s XAI governance layer produces auditable records compatible with EU AI Act transparency reporting requirements. The CTC Agent’s quality scoring rubric provides a standardized, documented assessment of explanation fidelity for every clinical output. The orchestration layer’s activation plans are logged and reproducible, supporting the traceability requirements of regulatory submissions. RAG-based knowledge grounding in all three agents reduces hallucination rates and supports factual reliability claims that would be required in device submissions (Neha et al., 2025). Data governance incorporating HIPAA and GDPR compliance through blockchain-based security architecture (Barbaria et al., 2025) addresses the data protection requirements that apply to any AI system processing personal health information. The call from international regulatory science researchers for urgent innovation in governance frameworks specifically designed for non-deterministic, broadly functional LLM-based medical devices (Ong et al., 2026; Armoundas and Singh, 2026; Meskó and Topol, 2023) provides the regulatory science context within which this framework is offered as a design contribution.

## 6 Limitations and future directions

This study represents a primary conceptual and exploratory initiative; therefore, it does not include empirical datasets for statistical validation, and its conclusions are intended to guide future research and clinical implementation rather than provide definitive evidence.

The primary limitation of this work is conceptual in nature. The framework has not been empirically validated, and the transition from design specification to validated clinical tool requires multi-institutional collaboration, clinical co-design with oncologists, computational implementation, and prospective evaluation. The framework should be read as a research agenda rather than a deployable system. Empirical validation of each agent is a necessary next step that will involve substantial resources, iterative refinement, and regulatory engagement.

Second, the choice of specific XAI methods for each agent reflects the current evidence base but should not be treated as permanent. The XAI landscape is evolving, and the modular architecture is designed to accommodate method replacement. Method transitions require their own validation, however, because changes to the explanation method change what clinicians see and may alter clinical decisions even when the underlying prediction model is unchanged.

Third, fairness deserves dedicated research attention independent of performance benchmarking. LLM-based oncological tools exhibit racial and gender biases documented in the literature (Agrawal, 2024), and lifestyle data from epidemiological studies systematically overrepresents high-income Western populations (Ktena et al., 2024; Marchesi et al., 2025). Bias auditing must be built into the validation protocol of each agent as a primary endpoint, not a secondary consideration. Generative AI approaches to dataset augmentation for underrepresented groups offer a partial mitigation strategy but do not substitute for demographic diversity in training data.

Fourth, clinical co-design of the CTC Agent’s explanation quality rubric is essential and requires structured engagement with oncologists, radiologists, and pathologists across cancer types and institutional contexts. The CLIX-M checklist provides the most developed precedent (Brankovic et al., 2025), but its adaptation to specific clinical scenarios in oncology has not been conducted. Future research should prioritize mixed-methods evaluation protocols in which clinician ratings of explanation usefulness are recorded prospectively during real clinical decisions and correlated with downstream patient outcomes.

Fifth, the causal interpretation framework embedded in the LRS Agent requires validation against known causal effect estimates from Mendelian randomization studies and randomized trials of lifestyle intervention in cancer prevention. The RAG knowledge base grounding the causal annotations must be regularly updated as new causal evidence emerges, requiring version control and systematic review protocols comparable to those applied to clinical practice guidelines.

Sixth, the economic implications of the proposed framework have not yet been formally evaluated. Because the framework remains conceptual and has not undergone prospective clinical deployment, robust cost-effectiveness analyses are currently not possible. Nevertheless, the LRS Agent’s Population-Level Preventive Summary component may offer important health-economic benefits by identifying modifiable cancer risk factors at the population level and supporting targeted prevention strategies. Earlier identification of high-risk groups and more efficient allocation of preventive interventions could potentially reduce downstream diagnostic and treatment costs associated with advanced-stage cancers. Future work should therefore incorporate formal health-economic evaluation using quality-adjusted life years (QALYs), incremental cost-effectiveness ratios (ICERs), and budget impact analyses alongside clinical validation studies.

## 7 Conclusion

This review documented four critical structural failures in the current XAI landscape for cancer diagnostics and proposed a three-agent framework designed to address them directly. Clinician exclusion, absent standardization, incomplete cross-omics explanations, and the near absence of lifestyle-driven XAI models are not peripheral technical problems; they are the core reasons that XAI has not delivered on its promise in oncological medicine despite nearly a decade of research investment and a rapidly expanding methods literature. Against this background, the acceleration of generative AI in oncology compounds the interpretability challenge: chain-of-thought reasoning does not constitute genuine transparency, hallucination has direct patient safety implications, and the deployment of powerful generative systems without formal XAI governance represents an unacceptable clinical risk. The research team’s multi-domain empirical record of evaluating LLMs in health contexts, spanning mental health assessment (Dergaa et al., 2024a), personalized nutrition (Dergaa et al., 2024b), exercise prescription (Dergaa et al., 2024c), and medical writing (Dergaa et al., 2024d), establishes that these limitations are structural rather than incidental and that formal oversight architecture is required for any clinical deployment.

The MO-XAI Agent targets the cross-layer interpretability gap by generating biologically attributed predictions that map SHAP outputs to specific pathway signatures and omics strata. The CTC Agent targets the clinician exclusion and standardization gaps by functioning as an explanation translation layer that evaluates XAI outputs against clinical quality standards before delivery and produces auditable quality records. The LRS Agent targets the public health gap by applying causal-annotated SHAP to modifiable lifestyle risk factors, grounding the resulting explanations in clinical epidemiology through RAG-augmented language modeling. Each agent addresses a distinct documented failure; together, they constitute a system-level response to failures that individual technical improvements have not resolved.

The framework was designed to be regulatorily aligned with the EU AI Act and FDA guidance for high-risk clinical AI from its first architectural decisions. Its implementation requires multi-institutional collaboration, clinician co-design, and prospective validation with a primary focus on demographic fairness as well as predictive performance. The TCGA, UK Biobank, and institutional EHR datasets provide viable validation pathways. The research group’s established competencies in clinical AI infrastructure (Boussi Rahmouni et al., 2025), healthcare data governance (Barbaria et al., 2025), hybrid language model applications to cancer pathology text (Abdaoui et al., 2025), and systematic evaluation of generative AI in clinical contexts (Dergaa et al., 2024a; Dergaa et al., 2024b; Dergaa et al., 2024c; Dergaa et al., 2024d; Dergaa et al., 2023; Dergaa et al., 2024e; Washif et al., 2024; Methnani et al., 2023) position this conceptual framework for transition into an empirical research program. Cancer diagnostics need AI systems that are accurate and trustworthy, powerful and governable, and technically sophisticated while remaining clinically co-designed. Among the three agents proposed, the CTC Agent carries the most immediate translational value because it can operate on existing XAI-equipped systems without requiring architectural changes. The MO-XAI Agent addresses the deepest scientific gap, and the LRS Agent offers the greatest public health return. Empirical validation of all three, with demographic fairness as a primary endpoint, constitutes the essential next step in this research program.

## Glossary

AI: Artificial Intelligence
CTC Agent: Clinician Trust and Communication Agent
CNVs: Copy Number Variations
EHR: Electronic Health Record
GANs: Generative Adversarial Networks
GenAI: Generative Artificial Intelligence
GNN: Graph Neural Network
GNN Explainer: Graph Neural Network Explainer
GO: Gene Ontology
Grad-CAM: Gradient-weighted Class Activation Mapping
GDPR: General Data Protection Regulation
HIPAA: Health Insurance Portability and Accountability Act
KEGG: Kyoto Encyclopedia of Genes and Genomes
LIME: Local Interpretable Model-Agnostic Explanations
LLM: Large Language Model
LRS Agent: Lifestyle-Driven Cancer Risk Stratification Agent
MO-XAI Agent: Multi-Omics XAI Agent
RAG: Retrieval-Augmented Generation
SHAP: SHapley Additive exPlanations
SurvSHAP: Survival SHAP
TCGA: The Cancer Genome Atlas
TNM: Tumor-Node-Metastasis
UK Biobank: United Kingdom Biobank
XAI: Explainable Artificial Intelligence
XGBoost: Extreme Gradient Boosting
CLIX-M: Clinician-informed XAI Evaluation Checklist with Metrics
FDA: Food and Drug Administration
EU AI Act: European Union Artificial Intelligence Act
JMIR: Journal of Medical Internet Research
GPT: Generative Pre-trained Transformer
ICCV: International Conference on Computer Vision
ACM SIGKDD: Association for Computing Machinery Special Interest Group on Knowledge Discovery and Data Mining
NPJ: Nature Partner Journals
WIREs: Wiley Interdisciplinary Reviews
